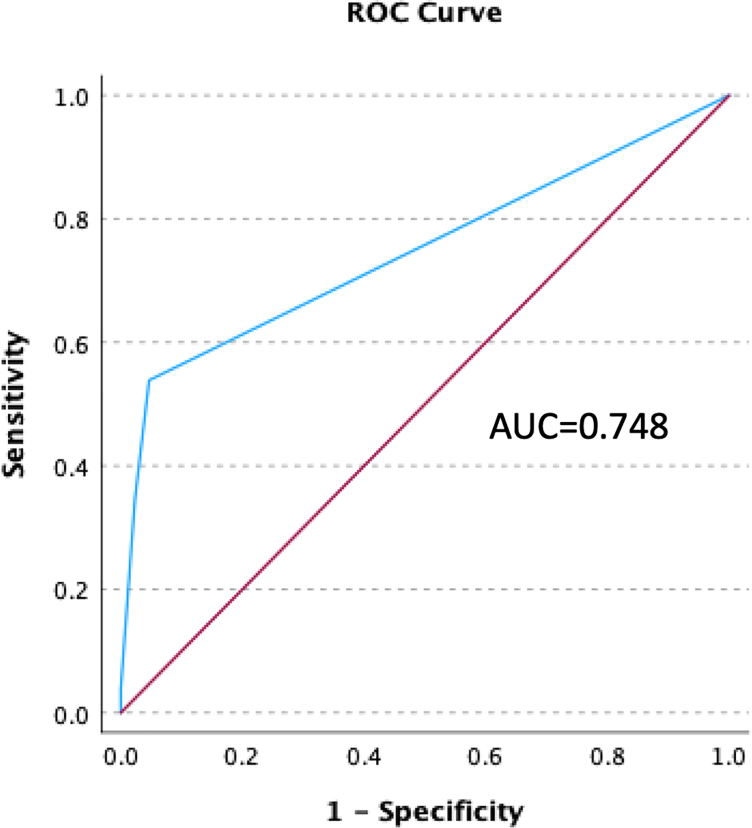# Electrocardiographic correlates of ventricular arrhythmias in repaired congenital heart disease

**DOI:** 10.1016/j.hroo.2024.10.006

**Published:** 2024-10-22

**Authors:** Satoshi Kawada, Hiroshi Morita, Koji Nakagawa, Tomofumi Mizuno, Takuro Masuda, Akira Ueoka, Saori Asada, Masakazu Miyamoto, Norihisa Toh, Nobuhiro Nishii, Shingo Kasahara, Shinsuke Yuasa

**Affiliations:** 1 Department of Cardiovascular Medicine, Okayama University Graduate School of Medicine, Dentistry and Pharmaceutical Sciences, Okayama, Japan; 2 Department of Cardiovascular Medicine, Kochi Health Sciences Center, Kochi, Japan; 3 Department of Cardiovascular Therapeutics, Okayama University Graduate School of Medicine, Dentistry and Pharmaceutical Sciences, Okayama, Japan; 4 Department of Cardiovascular Surgery, Okayama University Graduate School of Medicine and Dentistry, Okayama, Japan

**Keywords:** Congenital heart disease, Electrocardiogram, Implantable cardioverter defibrillator, Sudden cardiac death, Ventricular arrhythmias

## Abstract

**Background:**

Sudden cardiac death (SCD) is a major cause of mortality in congenital heart disease (CHD). Ventricular arrhythmias (VAs) are responsible for 80% of SCD. However, the clinical characteristics of patients with CHD and VAs are not fully understood.

**Objective:**

This study was undertaken to evaluate clinical and electrocardiographic characteristics of patients with CHD based on the presence or absence of VAs.

**Methods:**

We identified 28 patients with CHD with VAs (men, n = 15; 35.9 ± 16.5 years). Two patients with channelopathies were excluded from the analysis.

**Results:**

The most common diagnoses in patients with VAs were a transposition of the great arteries (n = 8, 30.8%), followed by tetralogy of Fallot (n = 7, 26.9%). Compared with patients without VA (n = 43), those with VAs had a longer QRS (160.6 ± 52.9 ms vs 133.4 ± 29.3 ms; *P* = .004), a higher prevalence of fragmented QRS (65.4% vs 37.2%; *P* = .022) and more epsilon wave (23.1% vs 2.3%; *P* = .005). Multivariable analysis showed that QRS > 180 ms (odds ratio [OR], 22.23; 95% confidence interval [CI], 2.40–206.53) and epsilon wave (OR, 14.33; 95% CI, 1.47–139.58) were significantly associated with VAs. During a median follow-up duration of 5.4 years, 5 patients (19.2%) received appropriate ICD therapy, and 3 patients (11.5%) died of heart failure.

**Conclusion:**

The assessment of QRS duration and the presence of fQRS and epsilon waves in patients with CHD could improve risk prediction for the development of VAs. These electrocardiogram (ECG) features could assist in clinical decision-making and might consequently reduce mortality.

## Graphical abstract

**Figure:**
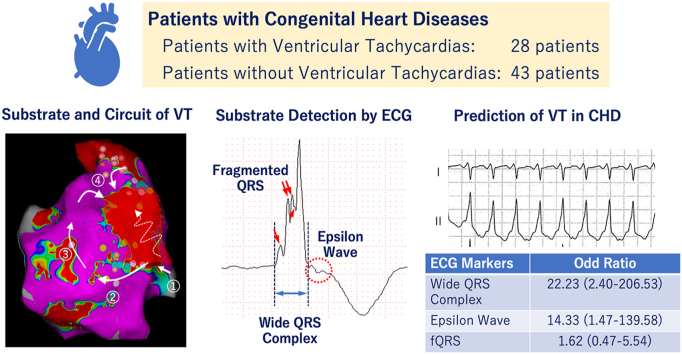

**Key findings**

▪ Our retrospective study showed that a wide QRS complex (>180 ms) and epsilon waves were significantly associated with ventricular arrhythmias (VAs) in patients with repaired congenital heart disease (CHD).
▪ Fragmented QRS was also associated with VAs.
▪ These ECG makers represent a significantly injured myocardium with conduction delay associated with the substrates for reentrant tachycardia.
▪ Assessment of the QRS duration and epsilon wave in patients with repaired CHD could improve risk stratification for the development of VAs and could assist in clinical decision-making and might consequently reduce mortality.

## Introduction

With the improvement of surgical techniques and medical therapy, most children with heart defects are now surviving into adulthood. Despite these medical achievements, ventricular arrhythmias (VAs) and sudden cardiac death (SCD) are still well-known late consequences after the surgical repair of a variety of congenital heart diseases (CHD).^1^ Previous studies have shown that up to 23.0% of deaths in adults with congenital heart disease were caused by SCD.^2^^,^^3^ The annual incidence of SCD in the CHD population is relatively low (0.09%/year). However, the relative risk of SCD in CHD is substantial compared with the age-matched general population.^4^ Although the risk of SCD increases with the severity of the disease, patients with mild disease still have an unignorable risk of SCD.^3^

The identification of subjects deemed at high risk for SCD is challenging because of the relatively small number of patients and modest event rates. Several risk features related to VAs in patients with tetralogy of Fallot (TOF), including QRS prolongation, fragmented QRS (fQRS), and right ventricular (RV) dilatation have been reported.^5^ Because of initial descriptions in the 1970s of SCD and its relationship to VAs, most studies seeking risk factors for VAs or SCD have focused on patients with TOF.^6^ Consequently, there are a paucity of data about the clinical characteristics of patients with CHD and VAs. A greater knowledge of individual risk factors for VAs in patients with CHD may contribute to elucidating which patients may benefit from interventions such as implantable cardioverter defibrillator (ICD) and catheter ablation.

The epsilon wave represents delayed anisotropic activation in the RV free wall and right ventricular outflow tract (RVOT), which is consistent with extensive endocardial scarring.^7^ An epsilon wave is reported to present in approximately 10% to 35% of patients with arrhythmogenic cardiomyopathy.^8^^,^^9^ Despite the high prevalence of epsilon waves in arrhythmogenic cardiomyopathy, it is not pathognomonic of the disease, because they also can be seen in cases of cardiac sarcoidosis, acute myocardial infarction, and Brugada syndrome.^10^, ^11^, ^12^ We hypothesized that the epsilon wave could be identified in CHD patients with VAs as RV dilation and dysfunction, which are frequently seen because of volume overload and surgical intervention. This study aimed to examine the clinical characteristics of VAs, including the presence of the epsilon wave, fQRS, and duration of QRS in patients with CHD. We also assessed the outcomes after VA events, including the delivery of appropriate ICD therapies and the major cardiac events (MACE).

## Methods

### Patient population and study design

Records of patients with CHD and VAs referred to Okayama University Hospital between 2004 and 2023 were retrospectively reviewed. We identified 28 CHD patients who developed VAs. VAs were defined as sustained VAs, including monomorphic ventricular tachycardia (VT) and polymorphic VT, and ventricular fibrillation (VF). Two patients who had channelopathy (TOF with *RyR2* mutation and ccTGA with *KCNH2* mutation) exhibited polymorphic VTs were excluded from the analysis because these mutations are known to be pathologic. Finally, 26 patients with VAs and 43 control patients with CHD without VAs who underwent catheter ablation for atrial tachyarrhythmias at our hospital during the same timeframe were included.

Our aim was to investigate the clinical and electrocardiogram (ECG) characteristics of patients with CHD compared between those with vs those without VAs. This study was approved by the Ethics Committee on Human Research and Epidemiology of Okayama University. The medical records of all patients with CHD were examined for baseline characteristics, clinical presentation, and device clinic follow-up in compliance with the guidelines for human studies of the Ethics Committee of Okayama University. The research reported in this paper adhered to ethical principles consistent with the Declaration of Helsinki. All patients gave their written informed consent for using their medical records.

### Electrocardiogram recording and measurements

The standard 12-lead ECG with a 0–150 Hz filter performed most closely after or before VA events was retrieved to evaluate ECG parameters at 400% size on a liquid crystal display. The rhythm, rate, morphology, and duration of QRS were determined. Specific ECG markers, including fQRS, QT interval, and epsilon wave, also were evaluated. The definition of fQRS has previously been reported.^13^ In short, fQRS was defined as ≥3 R-waves/notches in the R/S complex (more than the typical 2 in a right bundle branch block [RBBB]) in ≥2 contiguous leads. In patients with QRS < 120 ms, QRS fragmentation was defined as an additional R wave (R’) or notch in the nadir of the S wave. We evaluate the extent of fQRS as the number of ECG leads with fQRS. Epsilon waves were defined as independent small waves aside from the major QRS complex on the ST segment in ≥2 contiguous leads.^14^

All ECGs were analyzed by 2 independent observers (S. K. and H. M.) who were blinded to patient characteristics and clinical data.

### Outcome analysis

Heart failure events, death of any cause, and ICD therapies were evaluated. Heart failure was defined as hospitalization because of progressive worsening of exercise tolerance and fluid retention. Appropriate ICD therapy was defined as antitachycardia pacing or shock because of VAs. Therapies were considered inappropriate when delivered for a rhythm other than VA (either shock or antitachycardia pacing, whichever came first). A MACE after VAs was defined as appropriate ICD therapy, heart failure, and death of any cause.

### Statistical analysis

Data were presented as the mean ± standard deviation or median with interquartile range for continuous variables, depending on the normality of distributions on histograms. A Student *t* test or the Mann–Whitney *U* test was used to compare continuous variables between groups. Categorical variables were presented as absolute values and proportions (%). Pearson’s test or Fisher’s exact test was used to compare categorical variables between groups. To identify the predictors of VAs, logistic regression analysis was used because of the small number of events and the limited sample size. Results are reported in odds ratios (OR) with 95% confidence intervals (CIs). All tests were performed using SPSS V. 29.0 software Mac OS version. *P* < .05 was considered statistically significant.

## Results

### Baseline patient characteristics and clinical diagnosis

**Figure 1:**
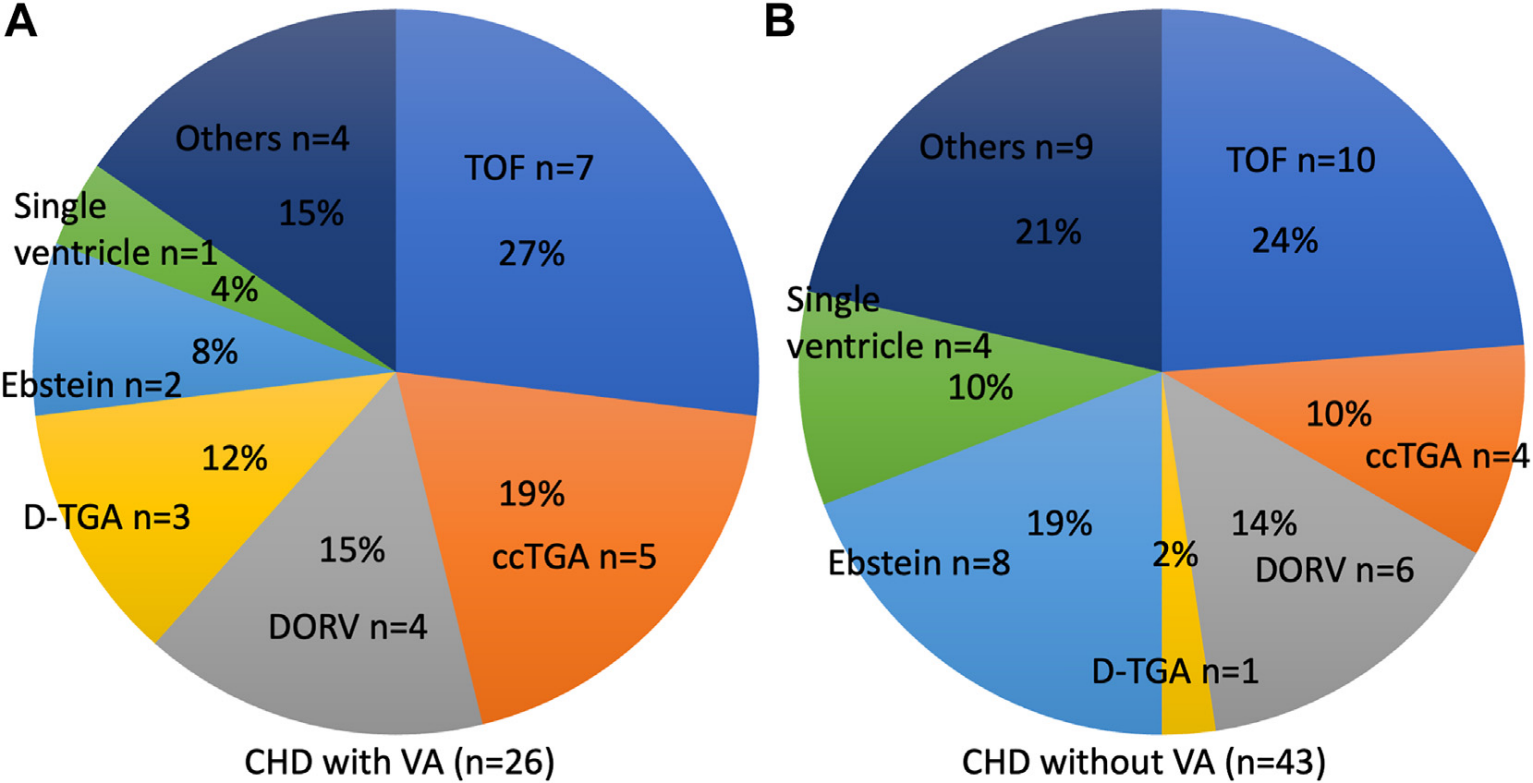
Details of cardiac defects. **A:** CHD cases with VA and **B:** CHD cases without VA. Others in the left panel (**A**) include AS n = 1, ALCAPA n = 1, HLHS n = 1, and VSD = 1. Others in the right panel (**B**) include AVSD = 2, TA = 3, and PA = 4. ALCAPA = anomalous left coronary artery from pulmonary artery; AVSD = atrioventricular septum defect; AS = aortic stenosis; ccTGA = congenitally corrected transposition of the great arteries; CHD = congenital heart disease; DORV = double-outlet right ventricle; D-TGA = dextro-transposition of the great arteries; PA = pulmonary atresia; TOF = tetralogy of Fallot; HLHS = hypoplastic left heart syndrome; VSD = ventricular septum defect.

A total of 26 patients with CHD and VAs, and 43 control patients without VA who underwent catheter ablation for atrial tachyarrhythmias referred to Okayama University Hospital from 2004 to 2023 were retrospectively reviewed. Approximately 90% of patients with VAs previously underwent cardiac surgeries. VT/VF occurred an average of 24.6 years after the first major cardiac surgery. The most common diagnosis of CHD in patients with VAs was a transposition of the great arteries (TGA) (n = 8, 30.7%), followed by TOF (n = 7, 26.9%), double-outlet right ventricles (DORV) (n = 4, 15.3%), and Ebstein’s anomaly (n = 2, 7.7%). TGA included dextro-transposition of the great arteries (D-TGA), using the Rastelli procedure with a systemic left ventricle (n = 3) and congenitally corrected transposition of the great arteries (ccTGA) (n = 5) (3 with a systemic left ventricle and 2 with a systemic right ventricle) (Figure 1). There was no significant difference between patients with and without VAs regarding the prevalence of systemic left and right ventricles in the 2-ventricle disease. Regarding patients with a single ventricle, 1 patient (3.8%) with a systemic left ventricle was identified in the VA group. Four patients (9.3%) (3 with a systemic left ventricle and one with a systemic right ventricle) were identified in the without VA group. Almost half of the patients (44.0%) with VAs had complex forms of CHD. Classification of CHD complexity was made based on the guideline.^15^ No significant differences were observed in the prevalence of Fontan circulation and severe complexity between the 2 groups. Amiodarone was used in 2 patients (7.7%), and a beta blocker was prescribed in 6 patients (24.0%) at the time of VAs. Systemic ventricular ejection fraction (EF) and the subpulmonary fractional area change (FAC) were not different between the patients with and without VAs. Laboratory data and medication history did not differ significantly between patients with CHD with and without VAs (Table 1). Baseline characteristics and details of cardiac defects with and without VAs are summarized in Table 1 and Figure 1.

**Table 1:** Baseline characteristics of patients with CHD with and without VA

	CHD with VA (n = 26)	CHD without VA (n = 43)	*P*
Sex (female)	42.3% (11/26)	44.2% (19/43)	.988
Age, y	37.5 ± 15.7	38.4 ± 19.6	.419
Body mass index	21.4 ± 4.6	22.6 ± 4.6	.151
Years after first major cardiac operation	25.4 ± 15.8	24.8 ± 12.0	.968
History of cardiac surgery, %	88.5% (23/26)	67.4% (29/43)	.051
Fontan circulation, %	12.0% (3/26)	27.9% (12/43)	.222
Severe complexity, %	44.0% (11/26)	51.2% (22/43)	.568
NYHA ≥ Ⅲ	13.3% (2/15)	15.0% (6/40)	.875
BNP, pg/mL	89.4 (56.6–171.2)	95.4 (55.8–285.9)	.970
Creatinine, mg/dL	0.97 ± 1.14	1.09 ± 1.21	.347
EF (subaortic), %	53.9 ± 12.8	56.6 ± 11.5	.200
FAC (subpulmonary), %	27.3 ± 12.8	34.5 ± 6.0	.081
AT/AF, %	50.0% (13/28)	100% (43/43)	N/A
Drugs at the time of VA occurrence or at ECG collection for those without VA			
Beta-blocker, %	24.0% (6/26)	48.8% (21/43)	.078
Antiarrhythmic drugs (excluding betablocker), %	30.8% (8/26)	16.7% (7/42)	.178
Amiodarone, %	7.7% (2/26)	16.7% (7/43)	.292
Digoxin, %	16.0% (4/25)	14.0% (6/43)	.819
Diuretics, %	57.7% (15/26)	100% (43/43)	N/A
Anticoagulation, %	42.3% (11/26)	100% (43/43)	N/A
Warfarin, %	34.6% (9/26)	39.5% (17/43)	.879

Data are expressed as a number (%) or as mean ± standard deviation.

AT/AF = atrial tachycardia and atrial fibrillation; BNP = B-type natriuretic peptide; CHD = congenital heart disease; ECG = electrocardiogram; EF = ejection fraction; FAC = fractional area change; NYHA = New York Heart Association; VA = ventricular arrhythmia.

### ECG characteristics in CHD patients with VAs

The ECG characteristics at the time of admission are described in Table 2. Compared with CHD patients without VAs, those with VAs had a longer QRS, a higher prevalence of fQRS, and more frequent epsilon waves. The prevalence of wide QRS complex (>180 ms) was significantly higher in patients with VAs compared with those without VAs. The 9 (32.1%) out of 28 patients with VAs had QRS > 180 ms, including those with TOF (n = 6), TGA (n = 2), and Ebstein’s anomaly (n = 1).

**Table 2:** The details of ECG characteristics of CHD with and without VA

	CHD with VA (n = 26)	CHD without VA (n = 43)	*P*
Rhythm			
Sinus rhythm	76.9% (20/26)	81.4% (35/43)	.656
Atrial fibrillation	0.0% (0/26)	2.3% (1/43)	N/A
Other rhythms	23.1% (6/26)	16.3% (7/43)	.488
PR interval, ms	224.4 ± 80.2	190.6 ± 51.8	.081
QRS duration, ms	160.6 ± 52.9	133.4 ± 29.3	.004
CRBBB, %	53.8% (14/26)	55.8% (15/43)	.874
Wide QRS complex (>180 ms)	34.6% (9/26)	2.3% (1/43)	.001
QT interval, ms	352 ± 62.1	349 ± 53.2	.909
Fragmented QRS, %	65.4% (17/26)	37.2% (16/43)	.022
Extent of fQRS			
Without fQRS	34.6% (9/26)	62.8% (27/43)	.022
Moderate fQRS (≤4 leads)	46.2% (12/26)	20.9% (9/43)	.029
Severe fQRS (≥5 leads)	19.2% (5/26)	13.2% (7/43)	.754
Epsilon wave, %	23.1% (6/26)	2.3% (1/43)	.005
Positive SAECG, %	68.8% (11/16)	38.5% (5/13)	.101

Other rhythms included atrioventricular nodal rhythm, pacing (ventricular), or atrial flutter.

CHD = congenital heart disease; ECG = electrocardiogram; SAECG = signal averaged electrocardiogram; VA = ventricular arrhythmias.

**Figure 2:**
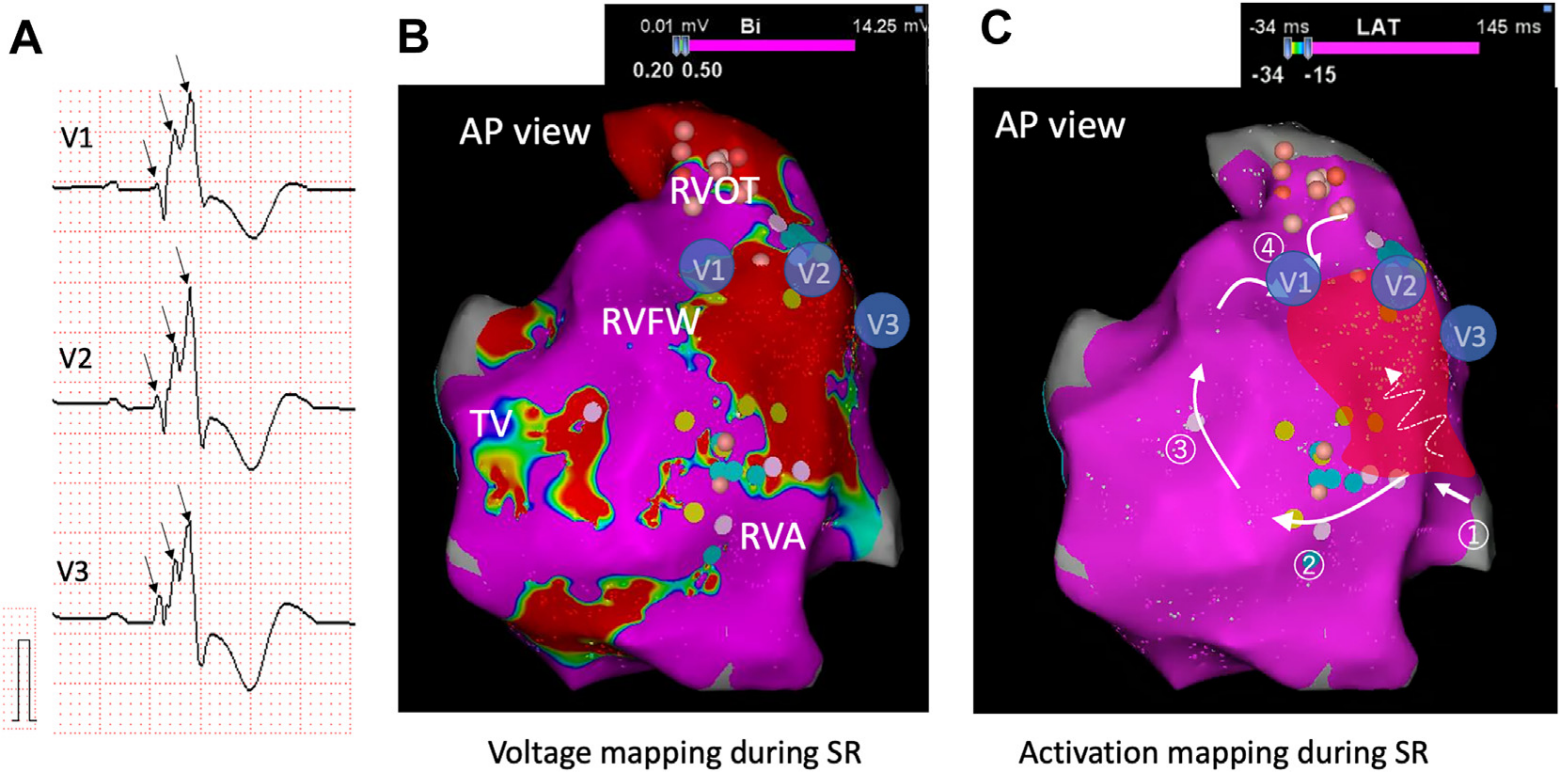
Changes of wavefront direction cause fragmented QRS. These figures were recorded in a 55-year-old woman with TOF who underwent RVOT reconstruction with a transannular patch and VSD closure. She had monomorphic VT with LBBB morphology and superior axis. **A:** A wide QRS complex with 3 positive spikes was seen in leads V1 to V3 during SR. **B:** Voltage mapping and **C:** activation mapping of RV was created during SR. Large low-voltage areas associated with surgical incisions were seen in RVOT and RVFW. V1 to V3 show hypothetical location of the precordial leads. Activation mapping showed changes of wavefront direction around the injured myocardium, representing fQRS in the surface ECG. The *circled numbers* represent order of propagation. Initial activation from the left ventricle ① caused the initial r waves in the ECG. Turn of the wavefront around the low-voltage area ② resulted in S wave, then the ascended excitation on the RV ③ caused nadir and a second r’ wave. At last, the circumnavigated excitation wavefront around the low-voltage area approaching the RVOT ④ constitute the last R” waves (Supplemental Video 1). VT related to a region of low-voltage area around RVFW was induced. After ablation targeting parts of reentrant circuits, VT was not inducible. ECG = electrocardiography; LBBB = left bundle branch block; RVOT = right ventricular outflow tract; RVFW = right ventricular free wall; SR = sinus rhythm; TOF = tetralogy of Fallot; VT = ventricular tachycardia.

Patients with VAs more frequently exhibited fQRS (65.4%) than did patients without VAs (37.2%). We also compared the extent of fQRS among patients with fQRS in both groups; however, no significant difference was found between CHD patients with and without VAs. High-density endocardial mapping in a patient with TOF and VAs showed changes of wavefront direction around the low-voltage area in the RV caused fQRS complexes in leads V1–V3 (Figure 2, Video 1 in the Supplemental material). In this case, VT related to a region of low voltage area in right ventricular free wall (RVFW) was induced, which was eliminated during ablation targeting these sites.

**Figure 3:**
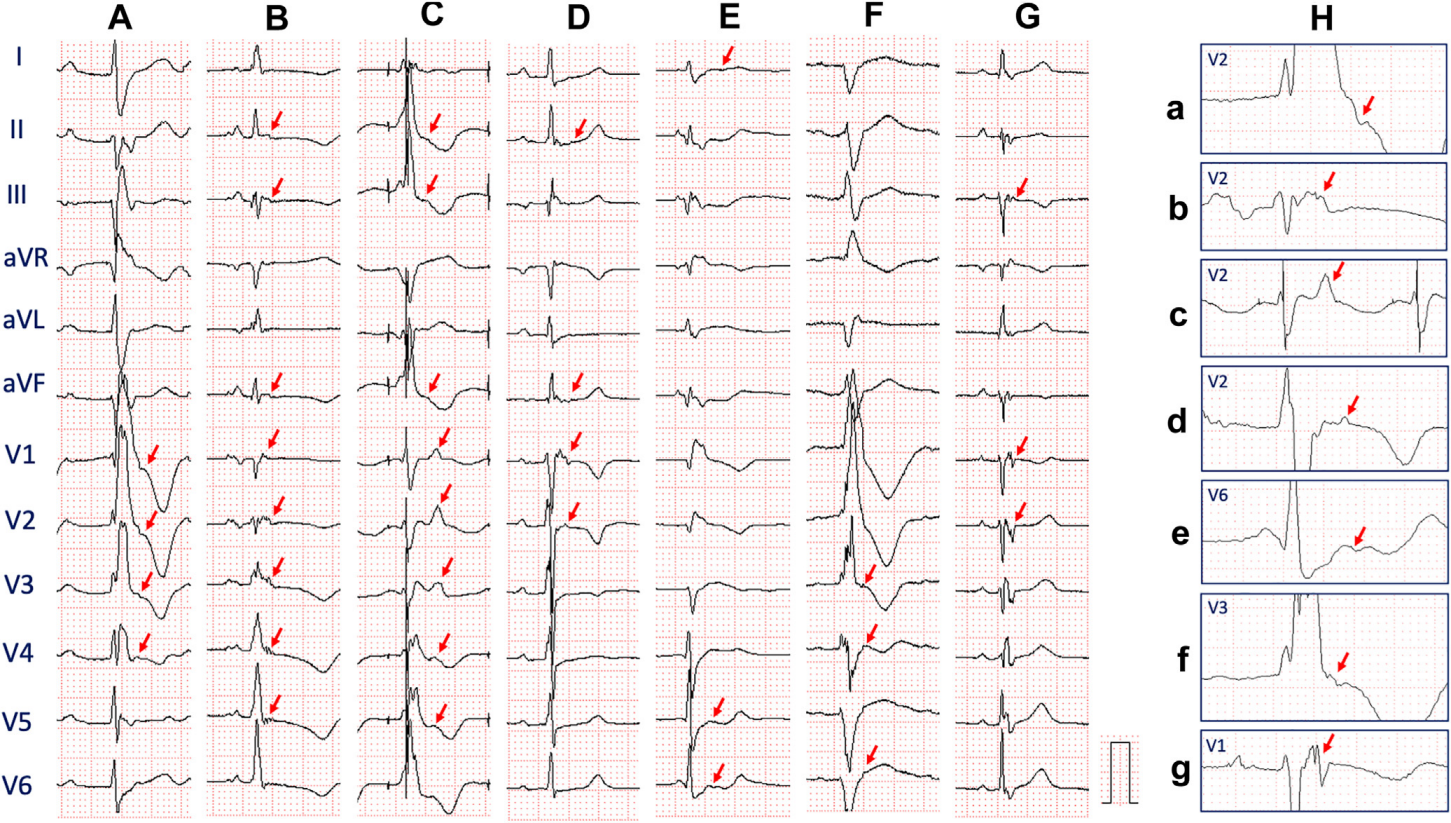
Twelve-lead ECG images of the patients with epsilon waves. Consecutive 12-lead ECG images of epsilon wave (**A**–**F:** CHD patients with VA; **G:** CHD patient without VA). Patient A: TOF, **B, C**, and **G:** Ebstein’s anomaly, **D:** D-TGA using the Rastelli procedure with a systemic left ventricle, **F:** cc-TGA with systemic right ventricle. **E:** DORV. **H:** Magnification of the leads with epsilon waves. a–g were from cases A–G, respectively. *Arrows* show epsilon waves after the major QRS complex. ccTGA = congenitally corrected transposition of the great arteries; CHD = congenital heart disease; DORV = double-outlet right ventricle; D-TGA = dextro-transposition of the great arteries; TOF = tetralogy of Fallot.

**Figure 4:**
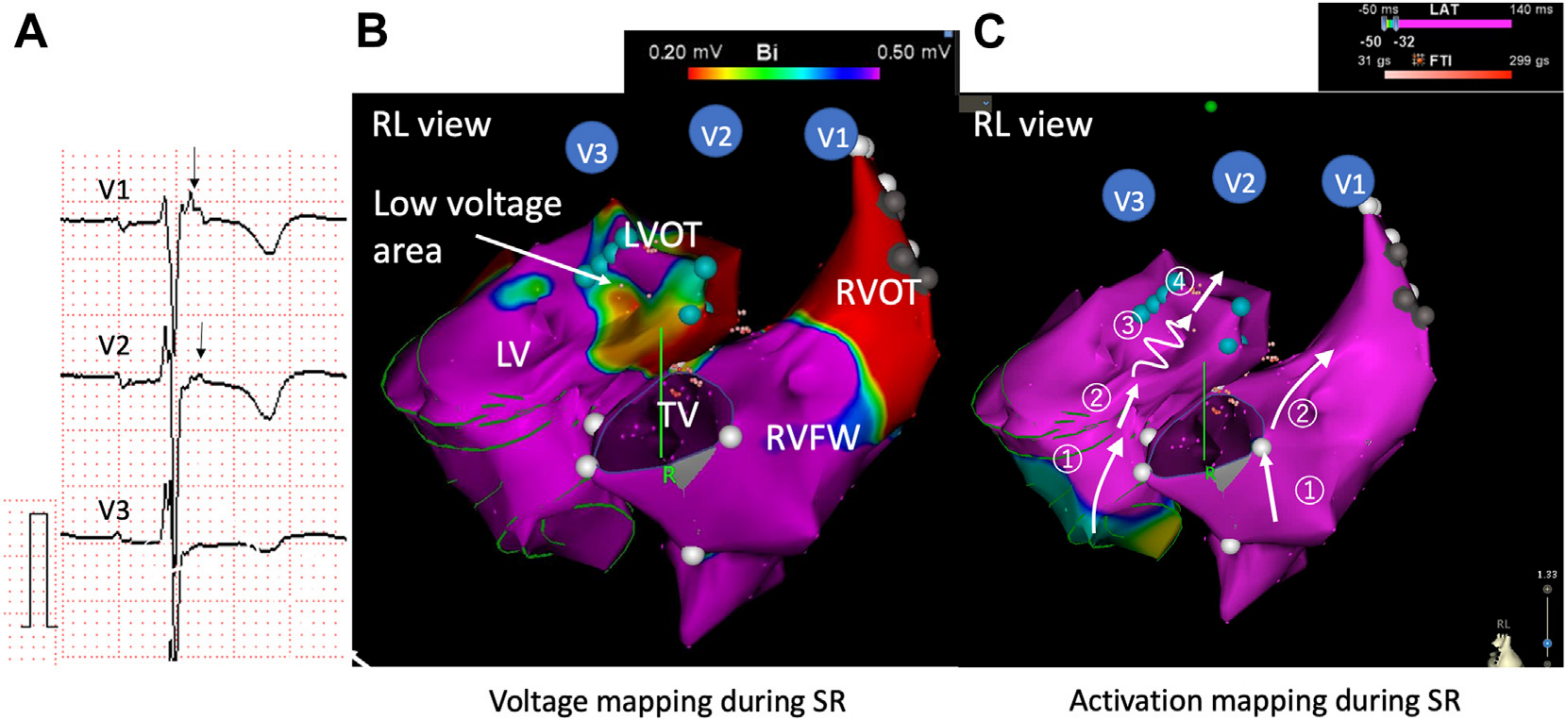
Significant-localized conduction delay causes epsilon wave. A 46-year-old woman with D-TGA/PS undergoing Rastelli procedure and LVOT reconstruction. She developed monomorphic VT with RBBB morphology and inferior axis. **A:** Narrow QRS with independent wave on the ST segment aside from QRS complex was seen as the lead V1–3 during sinus rhythm. **B:** Voltage mapping and **C**: activation mapping of both RV and LV was created during SR. Low-voltage areas were seen in LVOT during SR **(B)**. V1 to V3 show hypothetical location of the precordial leads. Activation mapping showed delayed conduction into the injured myocardium, which represented epsilon waves in the surface ECG. The number represents order of propagation (Supplemental Video 2). Sustained VT related to a region of abnormal potential including LVOT was induced. After catheter ablation targeting parts of reentrant circuits, VT was not inducible (**C**). D-TGA = dextro-transposition of the great arteries; ECG = electrocardiogram; LV = left ventricle; LVOT = left ventricular outflow tract; PS = pulmonary valve stenosis; RBBB = right bundle branch block; RV = right ventricle; VT = ventricular tachycardia.

The epsilon waves were found in 6 patients with VAs (23.1%) (TOF, n = 1; Ebstein’s anomaly, n = 2; TGA, n = 2; DORV, n = 1). Conversely, epsilon waves were identified in only 1 patient with Ebstein’s anomaly without VAs. Twelve-lead ECG images of epsilon waves in patients with and without VAs are shown in Figure 3. In a patient with dextro-transposition of the great arteries/pulmonary valve stenosis (d-TGA/PS) undergoing Rastelli procedure and left ventricular outflow tract (LVOT) reconstruction, high-density endocardial mapping showed that delayed conduction area surrounded by conduction block in the LVOT area created the epsilon waves in leads V1–V3 (Figure 4, Video 2 in the Supplemental material). In this case, VT related to the region of abnormal potential was induced. Ablation was successful in targeting parts of reentrant circuits, including LVOT.

There was no significant difference in the prevalence of sinus rhythm, positive signal averaged ECG, QT, or PR intervals between the 2 groups.

In patients with VAs, cycle lengths of VTs were 341 ± 105 ms. Monomorphic VTs occurred in 20 patients (71.4%), and polymorphic VT/VF was recorded in 8 patients (28.6%). More than half of the patients (64.2%) presented left bundle block (LBBB) morphology during VTs.

### The clinical course of CHD patients with VA

**Supplementary Figure 1:**
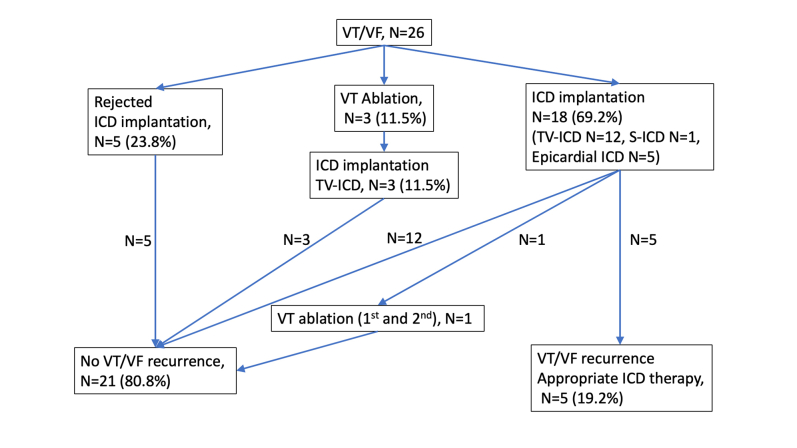


**Supplementary Figure 2:**
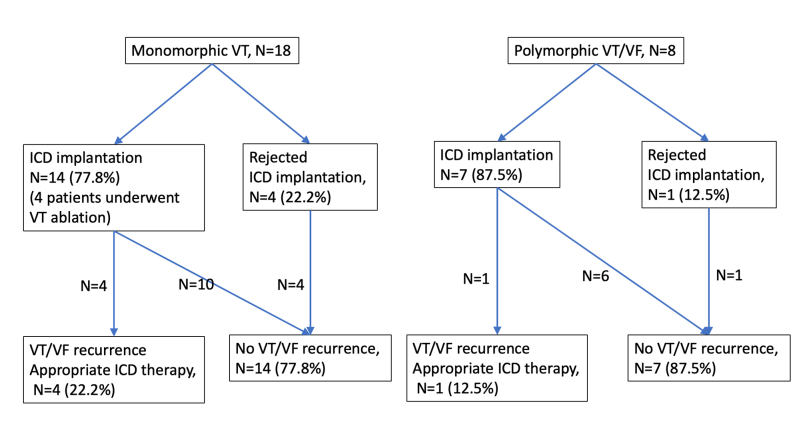


Finally, 23 patients (82.1%) received ICD (transvenous ICD, n = 15; subcutaneous ICD, n = 1; epicardial ICD, n = 7) after VAs. Five patients (17.9%) rejected ICD implantation. Three patients (11.1%) underwent catheter ablation for VT before ICD implantation. One patient (3.6%) underwent VT ablation twice after ICD implantation because of VT recurrence. Subsequently, all 4 patients who underwent VT ablation did not experience appropriate ICD therapy after the final ablation. During a median follow-up duration of 5.4 (4.1–10.6) years, 9 patients (32.1%) developed MACE. No clinical factors were significantly associated with MACE. In detail, 6 patients (21.4%) received appropriate ICD therapies, and 22 patients (78.6%) were free of VA recurrence. Seven patients (25.0%) received inappropriate ICD therapy during follow-up. Five patients (17.8%) were admitted with heart failure, and 3 of them (10.7%) died. No patient died because of VAs during follow-up. No clinical or ECG factors were significantly associated with MACE. Details of the clinical course of the patient population are shown in Supplementary Figures 1 and 2. Supplementary Tables 1 and 2 show the details of the patients with polymorphic VT/VF.

### ECG risk markers associated with VAs

Logistic regression analysis showed that wide QRS complex (>180 ms), fQRS, and epsilon waves were associated with VAs. Multivariable analysis showed that QRS > 180 ms (OR, 19.02; 95% CI, 2.09–173.46) and epsilon waves (OR, 12.25; 95% CI, 1.28–117.59) were significantly associated with VAs. Based on the outcomes of logistic regression analysis, we calculated a risk prediction score consisting of the total number of abnormal ECG markers, including QRS > 180 ms (=2 points) and epsilon wave (=1 point) (range, 0–3 points). The VA events increased as a score increased (OR, 7.12/1-point increase; CI, 2.27–22.4) (Table 3). The performance of the risk prediction model was examined using receiver operating characteristic (ROC) curves, which showed good discrimination (ROC = 0.748) (Supplementary Figure 3).

**Table 3:** Predictors of VA in patients with CHD

	Univariable	*P*	Multivariable	*P*
	Odds ratio		Odds ratio	
PR interval	0.99 (0.98–1.01)	.068		
Wide QRS complex (>180 ms)	22.24 (2.61–189.3)	.005	22.23 (2.40–206.53)	.006
fQRS	3.19 (1.15–8.82)	.026	1.62 (0.47–5.54)	.446
Epsilon wave, %	12.60 (1.42–111.79)	.023	14.33 (1.47–139.58)	.022
Positive SAECG, %	3.52 (0.76–16.39)	.109		
EF (subaortic), %	1.02 (0.98–1.06)	.394		
∗Risk prediction score (per an ECG marker increase)	7.12 (2.27–22.4)	.001		

Risk prediction score consists of total number of abnormal ECG markers including QRS > 180 ms (=2 points) and epsilon wave (=1 point) (range, 0–3).

CHD = congenital heart disease; EF = ejection fraction; SAECG = signal averaged electrocardiogram; VA = ventricular arrhythmias.

## Discussion

To the best of our current knowledge, this is the first study to demonstrate the high prevalence of epsilon waves in CHD patients with VAs. Our retrospective study showed that an fQRS, a wide QRS complex (>180 ms), and epsilon waves were significantly associated with VAs in patients with CHD. Those ECG abnormalities represent significant delayed conduction as arrhythmogenic substrates for VTs. The assessment of QRS duration and the presence of fQRS and epsilon waves in patients with CHD could improve risk stratification for VAs, could assist in clinical decision-making, and might consequently reduce mortality.

### Mechanisms and significance of epsilon waves in patients with CHD

In the current study, we identified epsilon wave as a possible new surrogate marker for the development of VAs in patients with CHD. This abnormal ECG marker is widely studied in cardiomyopathies, including arrhythmogenic cardiomyopathy and cardiac sarcoidosis, and it was identified as an important marker in CHD populations. The epsilon wave was first identified by Fontaine et al^16^ in 1978. They termed the tiny signals observed during both endocardial mapping and surface ECG in leads V1–V3 *epsilon waves*. The epsilon wave in arrhythmogenic cardiomyopathy represents delayed anisotropic activation in the RV free wall and RVOT, which means extensive endocardial scar. An epsilon wave is reported to present in approximately 10% to 35% of patients with arrhythmogenic cardiomyopathy.^8^^,^^9^ Epsilon waves also have been described for coronary artery disease (CAD), infiltrative disease, and Brugada syndrome.^17^ The high prevalence of epsilon waves is simpler to demonstrate in arrhythmogenic cardiomyopathy, because abnormal conduction is related to the presence of fat and fibrosis compared with CAD and cardiac sarcoidosis. Figure 4 shows a case with activation mapping during sinus rhythm, and delayed conduction in the large areas of the injured myocardium of left ventricular outflow created epsilon waves in the surface ECG (Figure 4 and Supplemental Video 2). Electroanatomic mapping during sinus rhythm may aid in defining arrhythmic substrate in patients with epsilon wave. The incidence of epsilon waves is not high in CHD cases with VTs. However, it is reasonable that appearance of epsilon waves is a surrogate marker for the development of VA, because epsilon waves are associated with severe conduction delay because of extensive myocardial scarring at that site.

### The mechanism and significance of wide QRS in CHD

Several studies described the importance of QRS duration as a predictor of SCD and VAs in patients with CHD.^5^^,^^18^ Previous studies demonstrated that QRS prolongation >180 ms was a strong risk factor for malignant VAs in patients with TOF.^5^ The increased arrhythmic events and mortality related to wide QRS reflect significant underlying myocardial disease with an arrhythmic substrate. According to a previous report, QRS prolongation reflects RV dilation rather than RV hypertrophy, suggesting that the mechanical and electrical properties of the RV create the circuits of reentrant tachycardia.^5^ In our study, QRS > 180 ms was evident in 9 of 26 (34.6%) patients with VAs, and it was strongly associated with VAs compared with the control group. The most common diagnosis of patients with QRS > 180 ms was TGA (n = 9, 32.1%), which was comparable to results of a previous study.^19^ Figure 4 shows a patient with high-density mapping in whom the excitation wavefront circumnavigated around the low-voltage wide area in the RV, and the detour of the excitation also caused long total activation time of ventricle.

Surgical scars are also important substrates for developing VAs.^15^ A previous study reported that QRS prolongation before or after pulmonary valve replacement, and the absence of a reduction in QRS duration after pulmonary valve replacement, are major risks of adverse outcomes in patients with TOF. In the current study, 7 (77.8%) of 9 patients with QRS > 180 ms underwent surgical intervention before VA events, which could influence QRS prolongation.^20^ Koyak et al^18^ reported that increased QRS duration (per 10-ms increase) was also associated with SCD in adult patients with CHD.^18^ The evaluation of changes in QRS characters and functional parameters can help predict VAs.^19^ However, we did not assess time-dependent changes in QRS duration in our sample.

### Mechanisms and significance of fragmented QRS in CHD

The significance of fQRS was initially studied and compared with Q waves in patients with CAD undergoing nuclear stress tests. Das et al^21^ reported that fQRS was associated with myocardial conduction block caused by myocardial scar, which was an independent predictor of cardiac events.^21^ Previous studies showed that fQRS was observed in 51% to 64% of CHD cases with sustained VA, which is comparable to the results our study.^22^^,^^23^ The prognostic value of fQRS appears to be driven mostly by its strong positive association with SCD in TOF. A recent study showed that fQRS was related to regional RV dysfunction and myocardial fibrosis in TOF.^24^ Furthermore, fQRS has been associated with a larger atrialized ventricular volume and arrhythmic events in patients with Ebstein’s anomaly.^25^

Fragmented QRS can be generated from the change of wavefront direction by the presence of a significantly injured myocardium with islands of viable myocardial tissue interspersed in abundant fibrous tissue, facilitating the development of reentrant tachycardia.^26^ The current study also showed that fQRS appeared more frequently in patients with VAs than in the control group. In our representative case, activation mapping showed the circumnavigated excitation wavefront around the wide and injured myocardium, representing fQRS in the surface ECG (Figure 2, Video 1 in the Supplemental material). fQRS represents an abnormal conduction disturbance as well as a long QRS interval and epsilon waves. Creating electroanatomic mapping during sinus rhythm before VT induction seems to be useful for revealing the reentrant circuits.

### Long-term outcomes in patients with CHD and VA

In our study, 23 patients (82.1%) received ICD after VAs. Five (17.8%) and 7 patients (25.0%) received appropriate and inappropriate ICD therapy during median follow-up periods of 5.4 years, respectively. Previous study demonstrated a high rate of appropriate and inappropriate ICD shocks during follow-up in patients with CHD, which was comparable to our study.^27^ Their data suggest that patients with VAs also have advanced atrial disease and fibrosis. Three patients died of heart failure; however, no patient died because of VAs during the follow-up. No clinical factors were associated with MACE, suggesting that the prognosis of these patients is mainly related to underlying substrate and relevant hemodynamics because of their cardiac defects.

### Clinical implication

Assessment of the QRS duration, the presence of fQRS, and epsilon waves in patients with CHD could improve risk stratification for the development of VAs, assist in clinical decision-making, and consequently reduce mortality. These ECG markers represent an injured myocardium with significantly conduction delay associated with the substrates for reentrant tachycardia. Patients with CHD and VAs may benefit from prophylactic ICD therapy or a more definitive treatment in the form of catheter ablation or surgery.

## Limitations

The study inherits all limitations of a retrospective study design of a relatively small number of participants enrolled at a single center. Analyses were performed for CHD in general instead of per type of CHD because of the limited number of patients in each group. In this retrospective study, we adopted patients with CHD without VAs who underwent catheter ablation for atrial tachyarrhythmias during the same timeframe as the control group because registry data for CHD were not available. Therefore, we could not identify age- or diagnosis-matched patients for inclusion in this study. Although the differences in almost all parameters did not achieve statistical significance between the groups with and without VAs, discrepancies were noted in the prevalence of atrial arrhythmias and the use of diuretics and anticoagulation. These factors might have affected the calculated measures of association. Second, there is a heterogeneity in the type of CHD. Third, the data were acquired retrospectively, and the number, interval, and documentation of clinical visits varied between patients. Fourth, we did not evaluate time-dependent changes in QRS characters and functional parameters in this study, which might have influenced our outcomes. Fifth, the standard 12-lead ECG performed most closely after or before VA events (7 patients underwent before [mean, 19.2 ± 22.5 days] and 19 patients just after VAs events). The use of antiarrhythmic drugs during VAs events may have influenced the ECG characteristics in our sample. Finally, because of the retrospective design, cardiac imaging including cardiac magnetic resonance imaging was not available to identify a substrate responsible for the fQRS and epsilon wave.

## Conclusions

Conduction abnormalities including wide QRS interval, fQRS, and epsilon waves were frequently seen in patients with CHD and VAs. The presence of multiple ECG factors was associated with high risk of VAs. Point-score by adding these ECG markers could stratify the risk of VAs in patients with CHDs. The assessment of ECG features could assist in clinical decision-making and consequently reduce mortality.

## Clinical perspectives

A wide QRS complex (>180 ms) and epsilon waves were significantly associated with VAs in patients with CHD. Fragmented QRS was also associated with VAs. These ECG markers represent a significantly injured myocardium, with conduction delay associated with the substrates for reentrant tachycardia. Assessment of the QRS duration and epsilon wave in patients with CHD could improve risk stratification for the development of VAs and could assist in clinical decision-making and might consequently reduce mortality.

## Figures and Tables

**Supplementary Figure 3:**