# Supplementary material for: Electrocardiographic correlates of ventricular arrhythmias in repaired congenital heart disease
Source: Heart Rhythm O2. 2024 Oct 22;6(1):39–47. doi: 10.1016/j.hroo.2024.10.006 (PMC11993798; doi:10.1016/j.hroo.2024.10.006)
Supplement: Supplemental material [file mmc1.docx]

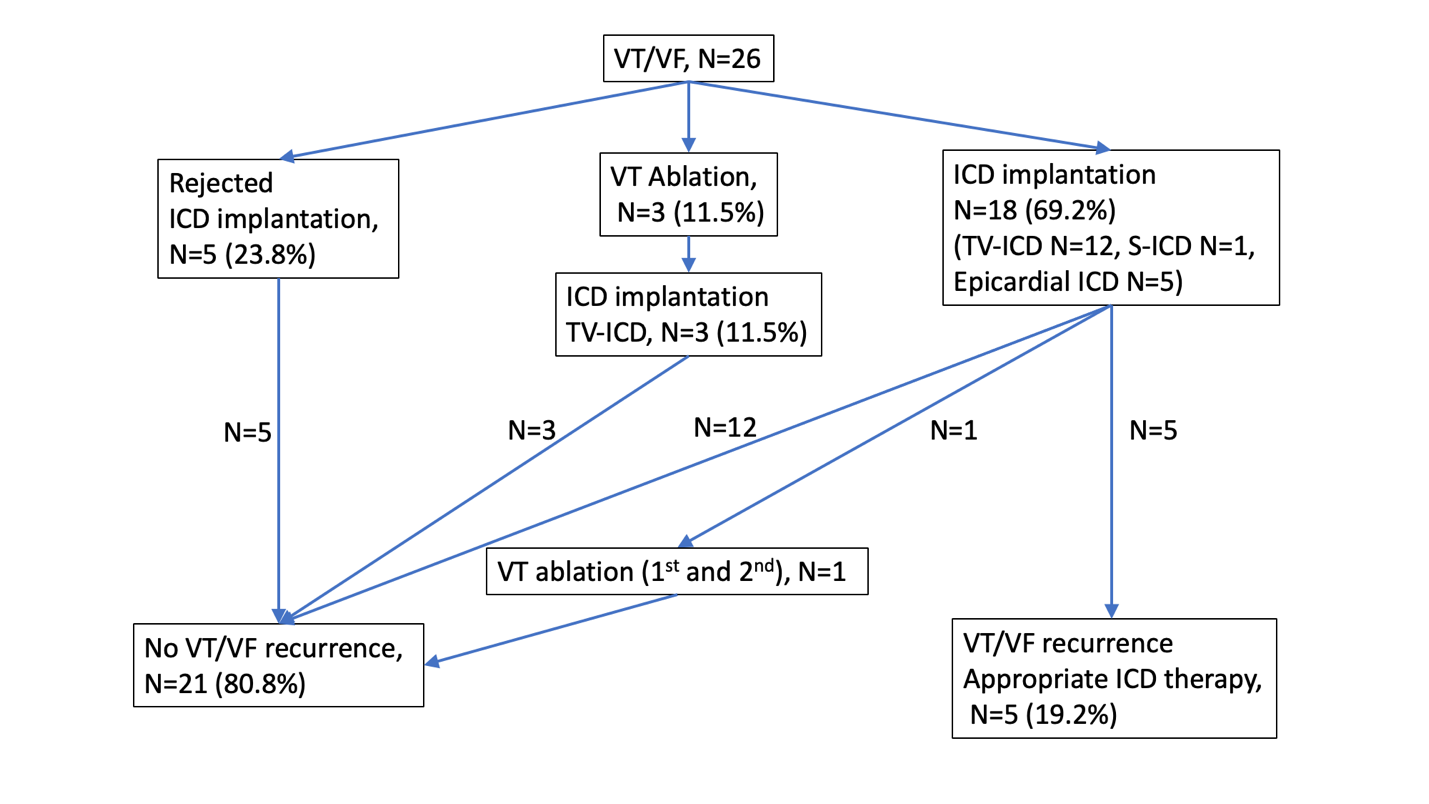
Supplementally figure 1. The clinical course of patients with CHD and VAs

TV-ICD, transvenous implantable cardioverter defibrillator; S-ICD; subcutaneous ICD; VT, ventricular tachycardia; VF, ventricular fibrillation


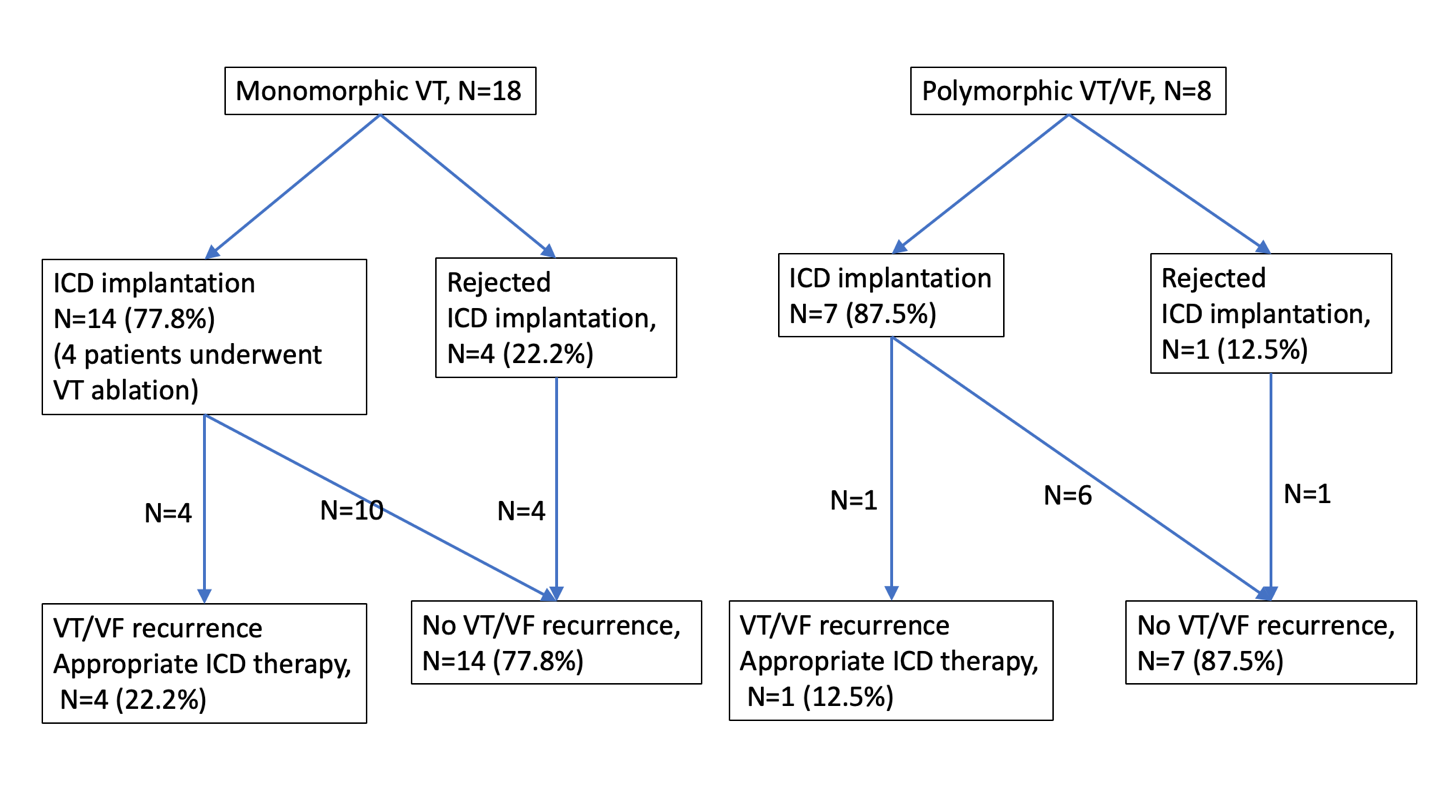


Supplementary figure 2. The more details of clinical course of patients with CHD and VAs,

ICD, implantable cardioverter defibrillator; VT, ventricular tachycardia; VF, ventricular fibrillation


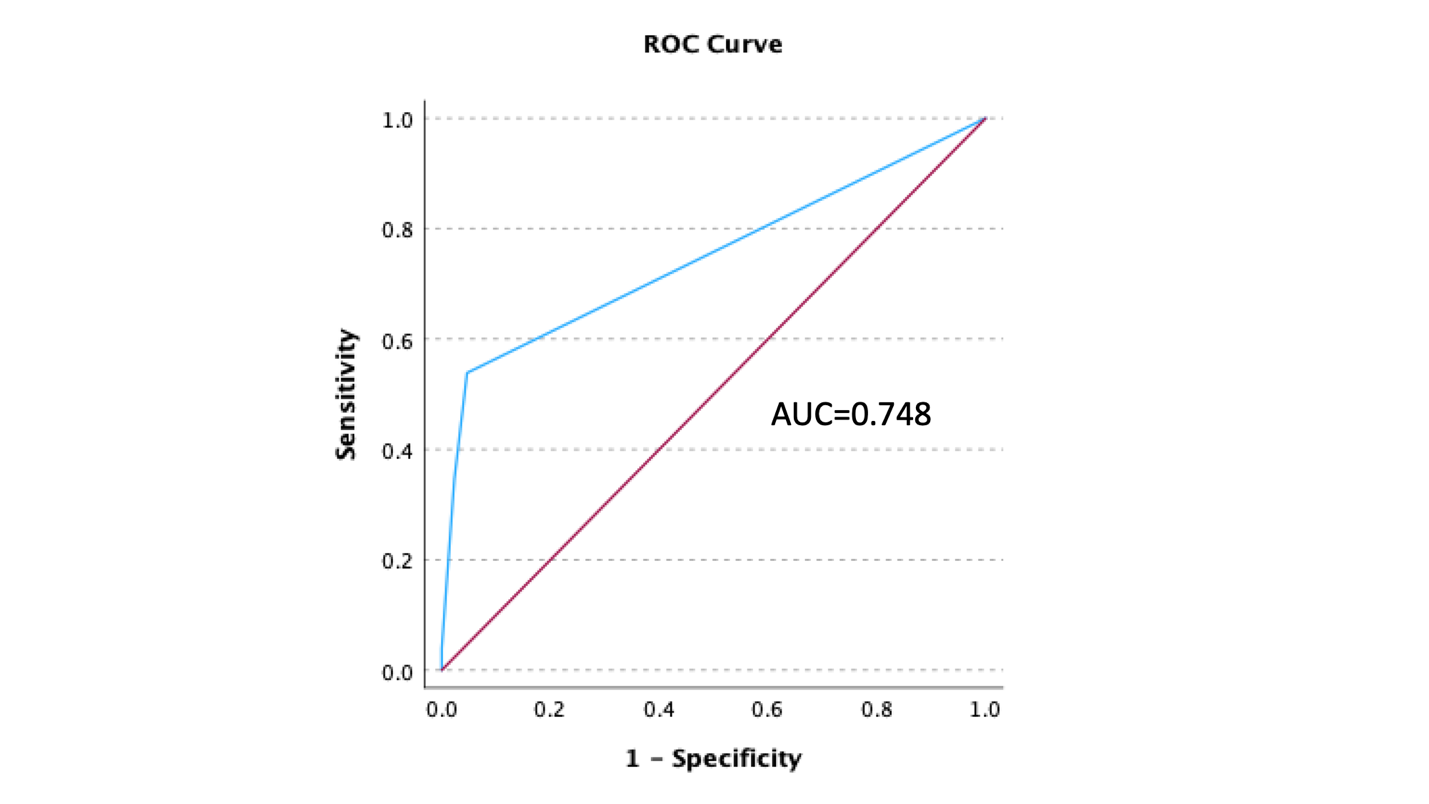


Supplementally figure 3. Receiver-operating characteristics curve of the risk prediction score.

The risk prediction score showed good discrimination (AUC=0.748). ROC curve confirmed 1.5 points as the best diagnostic cut-off value of score system for VAs (sensitivity of 90.0% and specificity of 34.6%).

Supplementary table 1. Baseline characteristics of the patients with polymorphic VT/VF.

| Diagnosis, years old, sex | Surgery | Years since surgery, years | BNP, pg/ml | EF (subaortic), % | NYHA | Medications at the time of VA |
| --- | --- | --- | --- | --- | --- | --- |
| TOF, 31 y,o Female | ROVT reconstruction with a transannular patch and VSD closure | 25.3 | 62.9 | 57 | Ⅱ | None |
| TOF, 66 y.o, Male | None | N/A | 1540 | 34 | N/A | ARB, digitalis and azosemide |
| ccTGA, 27 y.o, Female | TCPC | 26.5 | 45.2 | 70 | N/A | Carvedilol, furosemide and warfarin |
| ccTGA, 40 y.o, Female | Rastelli procedure and ASD, VSD patch closure | 24.8 | 166.3 | 49 | Ⅱ | Furosemide and warfarin |
| ccTGA, 17 y.o, Male | None | N/A | 8.4 | 48 | Ⅰ | none |
| HLHS, 9 y.o, Male | Modified Norwood | 8.9 | 24.9 | 53.3 | Ⅰ | Spironolactone, furosemide and aspirin |
| ccTGA, 22 y.o, Male | Double switch operation | 8.4 | 60.5 | 52 | Ⅰ | Warfarin |
| ALCAPA, 50 y.o, Female | LCA-Ao anastomosis, MV plasty | 1.0 | N/A | 45 | Ⅲ | Digitalis, spironolactone, furosemide, pilsicainide and aspirin |

Abbreviation as Figure 1

Supplementary table 2. The details of ECG characteristics and prognosis after ICD implantation in patients with polymorphic VT/VF.

|  | ECG at the time of VA occurrence | | |  |  |  | Events after VAs | |  |
| --- | --- | --- | --- | --- | --- | --- | --- | --- | --- |
| Diagnosis, years old, sex | Rhythm | PR interval (ms) | QRS duration (ms) | CRBBB | Fragmented QRS | Epsilon wave | Appropriate ICD therapies | Inappropriate ICD therapies | Heart failure events after VF events (admission) |
| TOF, 31 y,o Female | Sinus rhythm | 195 | 199 | Yes | None | None | Yes | Yes | None |
| TOF, 66 y.o, Male | Sinus rhythm | 166 | 186 | Yes | Yes | None | None | None | Yes |
| ccTGA, 27 y.o, Female | Sinus rhythm | 134 | 111 | No | None | None | None | None | None |
| ccTGA, 40 y.o, Female | Sinus rhythm | 123 | 144 | IVCD | None | None | N/A | N/A | Yes |
| ccTGA, 17 y.o, Male | Sinus rhythm | 111 | 108 | No | None | None | None | None | None |
| HLHS, 9 y.o, Male | Sinus rhythm | 131 | 96 | No | None | None | None | None | None |
| ccTGA, 22 y.o, Male | Sinus rhythm | 155 | 122 | Yes | None | None | None | Yes | None |
| ALCAPA, 50 y.o, Female | Sinus rhythm | 140 | 108 | Yes | None | None | None | Yes | None |

Abbreviation as Figure1. IVCD, interventricular conduction delay

Supplementary video 1. Activation mapping during sinus rhythm in a case of TOF with fragmented QRS (same case as figure 2)

Activation mapping showed changes of wavefront direction around the injured myocardium, representing fQRS in the surface ECG.

Supplementary video 2. Activation mapping during sinus rhythm in a case of d-TGA/PS with epsilon wave (same case as figure 4)

Activation mapping showed delayed conduction into the injured myocardium, which represented epsilon waves in the surface ECG.
